# Global Transcriptional Profiling of Granulosa Cells from Polycystic Ovary Syndrome Patients: Comparative Analyses of Patients with or without History of Ovarian Hyperstimulation Syndrome Reveals Distinct Biomarkers and Pathways

**DOI:** 10.3390/jcm11236941

**Published:** 2022-11-25

**Authors:** Maha H. Daghestani, Huda A. Alqahtani, AlBandary AlBakheet, Mashael Al Deery, Khalid A. Awartani, Mazin H. Daghestani, Namik Kaya, Arjumand Warsy, Serdar Coskun, Dilek Colak

**Affiliations:** 1 Department of Zoology, College of Science, King Saud University, Riyadh 11495, Saudi Arabia; 2 Department of Translational Genomics, Center for Genomic Medicine, King Faisal Specialist Hospital and Research Centre, Riyadh 11211, Saudi Arabia; 3 Department of Obstetrics and Gynecology, King Faisal Specialist Hospital and Research Centre, Riyadh 11211, Saudi Arabia; 4 Department of Obstetrics and Gynecology, Umm-Al-Qura University, Makkah 24382, Saudi Arabia; 5 Central Laboratory, Center for Women Scientific and Medical Studies, King Saud University, Riyadh 11451, Saudi Arabia; 6 Department of Pathology and Laboratory Medicine, King Faisal Specialist Hospital and Research Centre, Riyadh 11211, Saudi Arabia; 7 Department of Molecular Oncology, King Faisal Specialist Hospital and Research Centre, Riyadh 11211, Saudi Arabia

**Keywords:** ovarian hyperstimulation syndrome (OHSS), polycystic ovarian syndrome (PCOS), genome-wide gene expression, functional pathway, gene ontology, transcriptome, network analyses, biomarker

## Abstract

Ovarian hyperstimulation syndrome (OHSS) is often a complication of polycystic ovarian syndrome (PCOS), the most frequent disorder of the endocrine system, which affects women in their reproductive years. The etiology of OHSS is multifactorial, though the factors involved are not apparent. In an attempt to unveil the molecular basis of OHSS, we conducted transcriptome analysis of total RNA extracted from granulosa cells from PCOS patients with a history of OHSS (*n* = 6) and compared them to those with no history of OHSS (*n* = 18). We identified 59 significantly dysregulated genes (48 down-regulated, 11 up-regulated) in the PCOS with OHSS group compared to the PCOS without OHSS group (*p*-value < 0.01, fold change >1.5). Functional, pathway and network analyses revealed genes involved in cellular development, inflammatory and immune response, cellular growth and proliferation (including *DCN*, *VIM*, *LIFR*, *GRN*, *IL33*, *INSR*, *KLF2*, *FOXO1*, *VEGF*, *RDX*, *PLCL1*, *PAPPA*, and *ZFP36*), and significant alterations in the PPAR, IL6, IL10, JAK/STAT and NF-κB signaling pathways. Array findings were validated using quantitative RT-PCR. To the best of our knowledge, this is the largest cohort of Saudi PCOS cases (with or without OHSS) to date that was analyzed using a transcriptomic approach. Our data demonstrate alterations in various gene networks and pathways that may be involved in the pathophysiology of OHSS. Further studies are warranted to confirm the findings.

## 1. Introduction

Ovarian hyperstimulation syndrome (OHSS) is a rare, iatrogenic complication of ovarian hyperstimulation by assisted reproduction treatment and is potentially life-threatening [1,2,3]. Women with polycystic ovary syndrome (PCOS) are at a higher risk for developing OHSS due to having a large number of follicles on their ovaries and the tendency to over-respond to the hormones used for inducing fertility [2]. The causes of OHSS are generally unknown. Since complications can be severe and may even be life-threatening, it is critical to predict whether a woman going through gonadotropin therapy may develop OHSS.

Previous studies have reported several risk factors for the development of OHSS, such as young age, PCOS, low body mass index (BMI), and previous history of OHSS [4,5]. Granulosa cells (GC) are essential for ovarian folliculogenesis and are known to play a critical role in follicular development and oocyte maturation [6,7]. Several studies have indicated that GC dysfunction in women with PCOS may contribute to abnormal folliculogenesis [6,7]. Recent advances in omics technologies (genomics, transcriptomics, proteomics, and others) have enabled researchers to better understand the molecular characteristics of diseases and to identify disease biomarkers [7,8,9,10,11].

Genome-wide studies in various cells, including granulosa cells from women with PCOS and non-PCOS controls, have revealed several potential genes and pathways, such as the ERK/MAPK and VEGF signaling pathways [6,11]. However, OHSS (and PCOS) has a complex etiology and genetic basis and the disease pathogenesis and mechanism still largely remain unclear [5,6,12,13]. In this study, we aimed to identify the potentially essential genes and pathways in PCOS patients who would be prone to developing OHSS and to identify potential markers that may be linked to the OHSS and its pathobiology using a transcriptomic approach. To this purpose, we investigated if the PCOS patients who had developed OHSS have a distinct molecular profile in their granulosa cells as compared to those who did not in order to understand the biology of its development.

## 2. Materials and Methods

### 2.1. Patients

Twenty-four infertile women with PCOS undergoing IVF treatment were identified and examined by an IVF physician at Gynecology/IVF clinics at King Faisal Specialist Hospital and Research Center (KFSHRC). PCOS was defined according to the criteria established by the American Society of Reproductive Medicine and the European Society of Human Reproduction and Embryology (ASRM/ESHRE) [14]. The PCOS patients who are younger than 40 years, with FSH ≤ 12, body mass index (BMI) ≤ 35 kg/m^2^ and not undergoing any immunosuppressive therapy, were asked to participate in the study. The study was approved by the institutional review board (IRB) of the King Faisal Specialist Hospital and Research Center (no.08-MED604-2; RAC#2100002; RAC#2110006) and it is performed in accordance with the current version of the Helsinki Declaration. Written informed consent was obtained from all patients before participation in the study.

The study included samples from 24 patients (*n* = 24) with PCOS. Pituitary down-regulation was performed by administering gonadotrophin-releasing hormone (GnRH) agonist long or short protocols as described earlier [10]. Six patients had a positive history of OHSS (four patients had a history of OHSS and two had it in the current cycle) (referred to as “OHSS” group; *n* = 6) and the control group was without any history of OHSS (referred to as PCOS; *n* = 18). All OHSS cases were severe or moderate. Granulosa cells (3 mL) were collected into 15 mL regular falcon conical tubes from participating subjects undergoing control ovarian stimulation as described previously [10] for microarray and quantitative RT-PCR (qRT-PCR) experiments.

### 2.2. RNA Isolation

The total RNA was isolated from granulosa cells using Trizol or Total RNA Isolation Kit™ (ThermoFisher Scientific, Waltham, MA, USA) according to manufacturer’s instructions. The quality and quantity of RNA were determined by measuring absorbance spectra on a UV/Vis spectrophotometer, NanoDrop^®^ ND-1000 (ThermoFisher Scientific, Waltham, MA, USA). Further quality check was carried out using the RNA 6000 Nano Assay and 2100 Bioanalyzer (Agilent Technologies, Santa Clara, CA, USA). The high-quality RNA was either immediately used in the experiments (for the real-time RT-PCR and microarray experiments) or stored at −80 °C for further use.

### 2.3. Genome-Wide Gene Expression Profiling

Whole-genome gene expression profiling of samples from the OHSS (*n* = 6) and unrelated PCOS (*n* = 18) was performed using Affymetrix’s Human Genome U133 Plus 2.0 Array (ThermoFisher Scientific, Waltham, MA, USA). High-quality RNA was converted into labeled cRNA, fragmented, hybridized onto the chip surface according to the manufacturer’s protocols as described previously [15]. Briefly, the total RNA was converted into double-stranded cDNA, and then the cDNA was used for cRNA synthesis during which biotinylated UTPs and CTPs were incorporated into the cRNA. Target-labeled cRNA was fractionated and then hybridized onto the chip’s surface. The experimental procedures and quality control procedures at each critical step (before hybridization as well as post-hybridization) were strictly followed according to the manufacturer’s guidelines. Washing, staining, and scanning were performed according to the manufacturer’s instructions and guidelines.

Microarray data normalization was performed using GC Robust Multi-array Average (GC-RMA) algorithm [16,17]. We performed independent two-sample *t*-test to identify genes whose expression significantly varied between OHSS and PCOS groups. Differentially expressed genes (DEGs) were defined as those with *p*-value < 0.01 and fold change (FC) ≥ 1.5. Two-dimensional hierarchical clustering is performed using Pearson’s correlation with average linkage clustering. Functional annotation and biological term enrichment analysis were performed using DAVID Bioinformatics Resources [18]. Statistical analyses were performed using SPSS version 20 (SPSS, Inc., Chicago, IL, USA) and PARTEK Genomics Suite (Partek Inc., Chesterfield, MO, USA). All statistical tests were two-sided and *p*-value < 0.05 was considered statistically significant.

### 2.4. Functional, Pathway and Network Analyses

The functional, pathway, gene ontology enrichment, and network analyses were performed using Ingenuity Pathways Analysis (IPA) (QIAGEN Inc., Venlo, Netherlands; https://www.qiagenbioinformatics.com/products/ingenuity-pathway-analysis (accessed on 18 September 2020). The DEGs were mapped to their corresponding gene object in the Ingenuity Pathways Knowledge Base and significant gene interaction networks are identified. Scores of ≥2 were considered significant after applying a 99% confidence level. A right-tailed Fisher’s exact test was used to calculate a *p*-value determining the probability that the biological function (or pathway) assigned to that data set is explained by chance alone.

### 2.5. Quantitative RT-PCR (qRT-PCR)

To validate our microarray results, confirmatory qRT-PCR was performed using the ABI 7500 Sequence Detection System (ThermoFisher Scientific, Waltham, MA, USA). For this purpose, 50 ng total RNA procured from the same microarray study samples were transcribed into cDNA using Sensiscript Kit (QIAGEN Inc., Venlo, The Netherlands) under the following conditions: 25 °C for 10 min, 42 °C for 2 h, and 70 °C for 15 min in a total volume of 20 µL. Then, 2–5 μL of cDNA was amplified under the following conditions: Initial denaturation of 5 min at 95 °C followed by 34 cycles of “denaturation at 95 °C for 1 min, annealing at 60 °C for 1 min and extension at 72 °C for 1 min” and a final extension of 10 min at 72 °C.

Twenty-one genes (*FOXO1*, *FOXO3*, *FOXP1*, *GPC4*, *GPSM1*, *IL33*, *INSR*, *KLF2*, *MAN2B2*, *MYO10*, *NAGA*, *PAPPA*, *PCSK5*, *PHEX*, *PLD2*, *RABGAP1*, *SIN3A*, *SQSTM1*, *TCF7L2*, *USP9X*, and *ZFP36*) were randomly selected amongst the significantly expressed genes (*p*-value < 0.01) and primers were designed using Primer 3 web-toolsoftware (https://primer3.ut.ee accessed on 18 September 2022). The list of primer sequences used in this study is presented in Table S1. After primer optimization, the PCR assays were performed in 6 µL of the cDNA using the QIAGEN Quantitech SYBR Green Kit (QIAGEN Inc., Venlo, Netherlands), employing GAPDH as the endogenous control gene. All reactions were conducted in triplicates. The data were analyzed using the delta delta CT method [19,20].

## 3. Results

### 3.1. Clinical Characteristics of the Patients

The study included 24 women with PCOS with or without a history of OHSS. Granulosa cells were isolated from six PCOS patients with a history of OHSS (referred to as OHSS) and 18 without OHSS (referred to as the PCOS group). The clinical features were similar between the two groups of patients in terms of age, weight, BMI, FSH, LH, total testosterone, and androstenedione (Mann–Whitney U-test *p*-value > 0.05) (Table 1).

### 3.2. Identification of Differentially Expressed Genes

**Figure 1:**
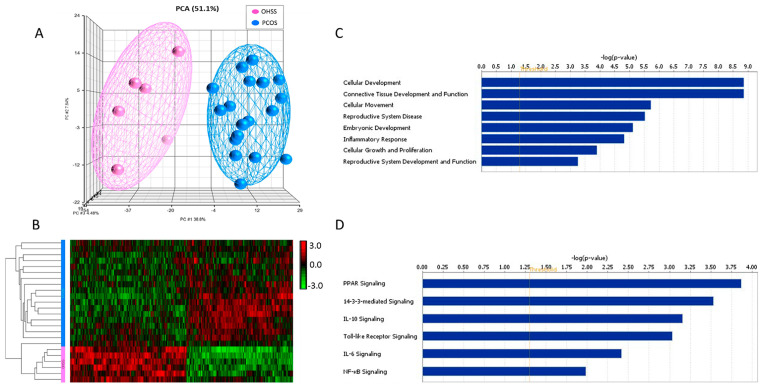
Global transcriptional changes associated with history of OHSS. (**A**) The unsupervised principal component analysis (PCA) and (**B**) two-dimensional hierarchical clustering analysis clearly distinguished individuals with PCOS with a positive history of OHSS from those without OHSS (**A**,**B**, respectively). The expression level of each gene across the samples is normalized to [−3, 3]. Hierarchical clustering was performed using Pearson’s correlation with average linkage clustering. Pink spheres indicate OHSS, blue spheres indicate PCOS (without OHSS). Red and green in the heatmap denote highly and weakly expressed genes, respectively. (**C**) Over-represented biological functions and (**D**) significantly altered canonical pathways associated with DEG (up- or down-regulated) in OHSS patients. *X*-axis indicates the significance (−log *p*-value) of the functional/pathway association that is dependent on the number of genes in a class as well as biological relevance. The threshold line represents a *p*-value of 0.05.

We analyzed the genome-wide mRNA expression profiling of 24 samples from OHSS and PCOS groups using Affymetrix’s GeneChip^®^ Human Genome U133 Plus 2.0 Arrays, which includes over 47,000 transcripts and variants using more than 54,000 probe sets. The array technology is a well-established and reliable method to assess global gene expression profiling [15,21]. Comparison of transcriptomes of OHSS with those PCOS patients with no history of OHSS revealed significant dysregulation of 1520 probes, corresponding to 1188 genes (*p*-value < 0.01), of which 65 probes (corresponding to 59 genes) had a greater than 1.5-fold change (FC) between the two groups (Table 2). The unsupervised principal component analysis (PCA) and hierarchical clustering in both dimensions (samples and genes) were performed using Pearson’s correlation with average linkage clustering, where both analyses revealed clear discrimination of samples as PCOS and OHSS as well as the pattern of genes deregulation defining two main transcriptome clusters (Figure 1A,B, respectively) that clearly demonstrated the significant differences in gene expression profiles of having a positive or negative history of OHSS among the PCOS patients.

### 3.3. Functional, Pathway and Gene Network Analysis of Dysregulated Genes

The gene ontology (GO) and functional analyses of differentially expressed genes in the OHSS group compared to PCOS were performed using IPA and DAVID Bioinformatics Resources [18,22]. The biological functions assigned to the data set were ranked according to the significance of the biological function to the dataset. As presented in Figure 1C, differentially expressed genes were enriched with functional categories including cellular development, connective tissue development and function, inflammatory and immune response, cellular growth and proliferation (including *DCN*, *FOXP1*, *GRN*, *IL33*, *INSR*, *KLF2*, *FOXO1*, *FOS*, *VIM*, *PAPPA*, and *ZFP36*). Significantly, altered canonical pathways in the OHSS included the PPAR, IL6, IL10, NF-κB and 14-3-3-mediated signaling pathways (Figure 1D).

**Figure 2:**
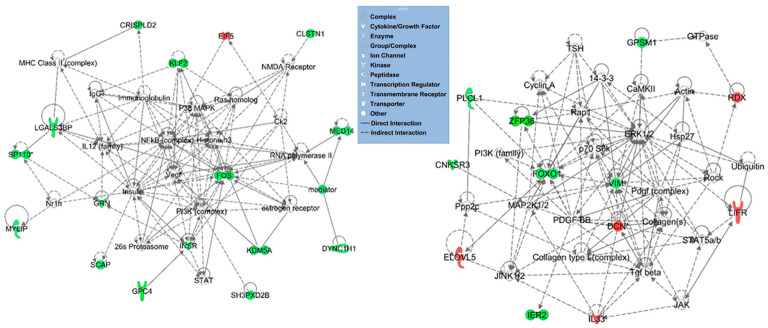
Gene interaction network analysis of significantly dysregulated genes in OHSS group. Top-scoring gene interaction networks with high relevancy scores are shown. Nodes represent genes and the edges indicate biological relationship between the nodes. Straight and dashed lines represent direct or indirect gene-to-gene interactions, respectively. The functional class of the gene product are represented with different shapes (see legend). Red/green indicated up- (down-) regulated in OHSS compared to PCOS group. The color intensity is correlated with fold change.

To gain an in-depth insight into the interactions of the dysregulated genes involved in the different pathways, genes that were significantly dysregulated in the OHSS were mapped to the gene networks using the Ingenuity Pathway Analysis [22,23]. The network analysis revealed potentially important hub genes, including *DCN*, *IL33*, *VIM*, *VEGF*, *GPC4*, *KLF2*, *ELOVL5*, *KDM5A*, Immunoglobulin, *ZFP36*, and the NF-κB, FOXO, and JAK/STAT signaling pathways that may be relevant to the pathophysiology of OHSS (Figure 2).

### 3.4. Confirmation of Gene Expression Using qRT-PCR

To validate the microarray results, confirmatory qRT-PCR was performed for randomly selected 21 significantly differentially expressed genes (*p*-value < 0.01), namely *FOXO1*, *FOXO3*, *FOXP1*, *GPC4*, *GPSM1*, *IL33*, *INSR*, *KLF2*, *MAN2B2*, *MYO10*, *NAGA*, *PAPPA*, *PCSK5*, *PHEX*, *PLD2*, *RABGAP1*, *SIN3A*, *SQSTM1*, *TCF7L2*, *USP9X*, and *ZFP36*, in OHSS compared to PCOS group (Figure S1). A strong correlation existed between the microarray and the qRT-PCR results (r = 0.9).

## 4. Discussion

In this study, we performed a transcriptomic comparison of PCOS patients with a history of OHSS to those patients with no history of OHSS using granulosa cells in order to identify potential markers for OHSS and to understand its biology. To the best of our knowledge, this is the largest cohort of Saudi PCOS cases (with or without OHSS) to date that was analyzed using a transcriptomic approach.

Our results revealed the potentially important roles of genes related to cellular development, connective tissue development and function, inflammatory and immune response, cellular growth and proliferation, and reproductive system development and function, including genes such as *FOXP1*, *FOXO1*, *DCN*, *IL33*, *INSR*, *KLF2*, *PAPPA*, *VIM*, and *ZFP36*, that have significant gene expression changes in the OHSS group compared to PCOS patients [24,25,26]. Forkhead box (FOX) transcription factor family members have critical aspects in modulating the genes that are important for various cellular processes such as cell growth, differentiation, and longevity and some of which have a crucial role in embryonic development [27], particularly in female reproduction [28]. Interestingly, Forkhead box P1 (*FOXP1*) is known to cause estrogen-dependent endometrial cancers through the KRAS pathway. Moreover, altered FOXP1 expression and Wnt-related β-catenin acetylation were observed in endometriotic stromal cells from endometriosis patients [29]. Knockdown experiments pointed out the dysregulation of genes involved in Wnt signaling and recapitulated the endometriotic cellular activities such as reducing collagen gel contraction and inhibiting cell proliferation [29]. Several studies have shown that the deletion of the *FOXO1* leads to embryonic cell death as a result of incomplete blood vessel development [30,31] and may have a critical role in placental morphogenesis in the developing embryo [32,33]. *FOXO1* was implicated in putatively regulating genes involved in lipid and sterol biosynthesis, suggesting that it may play a role in follicular steroidogenesis (Liu et al., 2009). Furthermore, the expression of *FOXO1* is shown to be associated with estrogen receptors alpha (ER-α) and beta (ER-β), which are produced primarily by the ovaries and the placenta during pregnancy. The follicle-stimulating hormone (FSH) stimulates the ovarian production of estrogens by the granulosa cells of the ovarian follicles and corpora lutea [34,35]. The pregnancy-associated plasma protein A (*PAPPA*) is significantly up-regulated in OHSS patients. *PAPPA* may have a role in female fertility by modulating ovarian function, preeclamptic placentae and steroidogenesis [24,36].

Zing finger protein (*ZFP36*), Kruppel-like factor 2 (*KLF2*), insulin receptor (*INSR*), elongation of very-long-chain fatty acid (*ELOVL5*) and Glypican-4 (*GPC4*) were found to be significantly altered in OHSS. *GPC4*, which is an adipokine that interacts with the INSR and influences insulin sensitivity [37], was down-regulated in OHSS, which may have a likely role in affecting the control of cell division, growth regulation, body fat distribution, insulin resistance, and arterial stiffness in OHSS [38]. Zing finger protein, *ZFP36*, was shown to be crucial for female fertility and early embryonic development [25] and influences ovulation and oocyte maturation [39,40]. Therefore, it was proposed as a promising candidate gene for obesity-associated metabolic complications [41]. Altered insulin functions have long been associated with abnormalities in female reproduction [42,43]. Conditions associated with insulin resistance, such as obesity and diabetes mellitus, are often accompanied by increased adiposity or hyperglycemia [44]. Obesity and diabetes are independently associated with an altered female reproductive function [13,45,46]. Interestingly, a recent study reported that *ELOVL5* is involved in embryonic development and lipid metabolism [47]. *KLF2* is expressed in endothelial cells and inhibited by the inflammatory cytokine interleukin-1, hence, implicated as a novel regulator response to proinflammatory stimuli [48]. KLF2′s inhibition for endothelial cell migration and angiogenesis is partly attributed to its ability to inhibit the VEGF receptor (VEGFR) 2/kinase insert domain-containing receptor (KDR) expression [49]. Moreover, G-protein signaling modulator 1 (GPSM1) and Myosin X (MYO10) also displayed dysregulation in OHSS patients. *GPSM1* plays a critical role in regulating mitotic spindle orientation, cell polarity, and adenylyl cyclase activity [50] and *MYO10* has key functions in filopodia. Experiments with fibroblast-like cells have revealed that MYO10 localizes to the tips of filopodia and undergoes intrafilopodial motility [51].

The pathway and gene network analyses revealed significant alterations in the PPAR, IL6, IL10, FOXO, JAK/STAT and NF-κB signaling pathways and potentially critical roles of *IL33*, *VEGF*, *INSR*, *FOS*, *TGf-β*, *LIFR*, and immunoglobulin that may be relevant to the pathophysiology of OHSS [52,53,54]. Indeed, previous studies also reported the involvement of the immune system, cytokines, and growth factors in the pathogenesis of OHSS [52,53,55,56]. For example, *VEGF* was implicated as having a significant role in the development of OHSS [26,52,54,57]. *IL33* acts as both an extracellular cytokine and an intracellular nuclear factor with transcriptional regulatory properties [58]. IL33 was shown to have higher levels in PCOS patients compared to the controls [59]. Interestingly, dehydroepiandrosterone (DHEA)-induced PCOS in rats was shown to respond to omega-6 fatty acid (γ-linolenic acid (GLA)) treatment that led to a significant decrease in IL-33 levels in the rat’s ovaries, hence, implicating GLA’s potential use for likely human-related treatments for inflammatory responses in PCOS via the PPAR-γ pathway [60]. Previous studies have reported that the JAK/STAT pathway plays a critical role in the regulation of functions including immune regulation, growth, fertility, and embryogenesis [61,62,63,64].

A limitation of this study was that we had a relatively small sample size within the OHSS group. As the occurrence of OHSS is quite low (about 5% of treated women may encounter moderate and severe OHSS), we were able to recruit six patients with a history of OHSS (*n* = 6) (four patients had a history of OHSS and two had it in the current cycle) and 18 without any history of OHSS (*n* = 18) for our whole-genome expression analysis. This was, indeed, achieved after several years of sample collection. For whole-genome studies of such rare human diseases, including PCOS/OHSS, this number is considered a reasonable number to be able to identify differentially expressed genes (DEGs) for discovery-related investigations [6,54,65]. In addition, we used a stringent cutoff for selecting the DEGs; a *p*-value (of less than 0.01) as well as the fold change criteria. Furthermore, we performed an independent experimental method (qRT-PCR) to validate the microarray results and there was a strong correlation that existed between the microarray and qRT-PCR results (r = 0.9). Future studies may focus on investigating functional mechanisms and further validating the findings on a larger group of patient cohorts.

## 5. Conclusions

In conclusion, in this study, we identified markers and altered pathways that may have the potential to differentiate the patients who may be prone to developing OHSS and understanding the biology of its development, which opens new avenues for further research to confirm these findings.

## Figures and Tables

**Table 1:** Comparison of clinical parameters between OHSS group and PCOS group.

	PCOS (*n* = 18)	OHSS (*n* = 6)	*p*-Value *
	Mean (SD)	Mean (SD)	
Age (years)	29.0 (3.7)	30.2 (6.3)	0.71
BMI (kg/m^2^)	24.8 (1.2)	24.2(1.4)	0.43
FSH (IU/L)	6.9 (2.2)	6.3 (1.1)	0.47
LH (IU/L)	13.0 (9.9)	7.5 (4.9)	0.14
Testosterone	2.5 (1.3)	2.2 (0.9)	0.76
Androstenedione	12.3 (6.8)	15.5 (4.8)	0.13

* Mann–Whitney U-test. BMI, Body mass index; FSH, Follicle stimulating hormone; LH, Lutenising hormone. OHSS refers to PCOS patients with a history of OHSS and PCOS refers to patients without any history of OHSS.

**Table 2:** Significantly altered probes in PCOS patients with history of OHSS as compared to no history of OHSS (*p*-value < 0.01 and >1.5-fold change).

Gene	Gene Title	*p*-Value	FC
*PAPPA*	pregnancy-associated plasma protein A, pappalysin 1	0.000798	1.86
*DCN*	Decorin	0.0044	1.76
*PTPLA*	protein tyrosine phosphatase-like (proline instead of catalytic arginine), member	0.005688	1.71
*FKBP1A*	FK506 binding protein 1A, 12 kDa	0.002428	1.7
*SHOX2*	short stature homeobox 2	0.008934	1.64
*ELOVL5*	ELOVL family member 5, elongation of long-chain fatty acids (FEN1/Elo2, SUR4/Elo)	0.000683	1.55
*IL33*	interleukin 33	0.003649	1.55
*EIF5*	eukaryotic translation initiation factor 5	0.007926	1.52
*TMX1*	thioredoxin-related transmembrane protein 1	0.000272	1.52
*LIFR*	leukemia inhibitory factor receptor alpha	0.00038	1.51
*RDX*	Radixin	0.005259	1.51
*OBSL1*	obscurin-like 1	0.009828	−1.5
*RNPEP*	arginylaminopeptidase (aminopeptidase B)	0.000196	−1.5
*BAT3*	HLA-B associated transcript 3	0.001025	−1.51
*DYNC1H1*	dynein, cytoplasmic 1, heavy chain 1	0.004793	−1.51
*FOXP1*	forkhead box P1	0.001132	−1.51
*MAP4K4*	mitogen-activated protein kinase kinasekinasekinase 4	0.000309	−1.51
*GRN*	Granulin	0.008224	−1.52
*MED14*	mediator complex subunit 14	0.001012	−1.52
*HS3ST1*	heparan sulfate (glucosamine) 3-O-sulfotransferase 1	0.007768	−1.53
*SEC14L1*	SEC14-like 1 (S. cerevisiae)	0.005745	−1.53
*SNRNP200*	small nuclear ribonucleoprotein 200k Da (U5)	0.001182	−1.53
*BBS1*	Bardet–Biedl syndrome 1	0.000834	−1.54
*MAN2B2*	mannosidase, alpha, class 2B, member 2	0.00033	−1.54
*MYLIP*	myosin regulatory light chain interacting protein	0.006807	−1.55
*KIAA0513*	KIAA0513	0.004853	−1.56
*KDM5A*	lysine (K)-specific demethylase 5A	0.000431	−1.57
*PCSK5*	proproteinconvertasesubtilisin/kexintype 5	0.002482	−1.58
*GAS2L1*	growth arrest-specific 2 like 1	0.005994	−1.59
*MGC21881*	hypothetical locus MGC21881	0.009274	−1.59
*PLCL1*	phospholipase C-like 1	0.009504	−1.59
*ZSWIM5*	zinc finger, SWIM-type containing 5	0.005745	−1.59
*VIM*	Vimentin	0.001645	−1.6
*CRISPLD2*	cysteine-rich secretory protein LCCL domain containing 2	0.00095	−1.61
*GPSM1*	G-protein signaling modulator 1	0.002004	−1.61
*CHST3*	carbohydrate (chondroitin 6) sulfotransferase 3	0.001192	−1.63
*SCAP*	SREBF chaperone	0.008736	−1.63
*LAMP1*	lysosomal-associated membrane protein 1	0.008512	−1.64
*SH3PXD2B*	SH3 and PX domains 2B	0.004433	−1.64
*SP110*	SP110 nuclear body protein	0.00000735	−1.66
*PHEX*	phosphate regulating endopeptidase homolog, X-linked	0.004347	−1.67
*FOXO1*	forkhead box O1	0.001054	−1.68
*TPP1*	tripeptidyl peptidase I	0.009215	−1.69
*LGALS3BP*	lectin, galactoside-binding, soluble, 3 binding protein	0.007244	−1.71
*ABCA8*	ATP-binding cassette, sub-family A (ABC1), member 8	0.004646	−1.76
*CADM1*	cell adhesion molecule 1	0.003501	−1.77
*CLSTN1*	calsyntenin 1	0.002802	−1.77
*FOS*	FBJ murine osteosarcoma viral oncogene homolog	0.00617	−1.81
*MYO10*	myosin X	0.004334	−1.86
*KLF2*	Kruppel-like factor 2 (lung)	0.003583	−1.87
*INSR*	insulin receptor	0.008567	−1.88
*IER2*	immediate early response 2	0.006056	−1.91
*CNKSR3*	CNKSR family member 3	0.004281	−1.99
*GPC4*	glypican 4	0.003565	−2.08
*MYADM*	myeloid-associated differentiation marker	0.002895	−2.14
*PXDN*	peroxidasin homolog (Drosophila)	0.00015	−2.15
*DAPK1*	death-associated protein kinase 1	0.001645	−2.17
*ZFP36*	zinc finger protein 36, C3H type, homolog (mouse)	0.000679	−2.38

FC, fold change between patients with history of OHSS as compared to PCOS patients. Negative sign indicates down-regulation.

## Data Availability

All data generated and analyzed in this study are included in this manuscript and its Supplementary Materials files.

## Supplementary Materials

The following supporting information can be downloaded at: https://www.mdpi.com/article/10.3390/jcm11236941/s1. Figure S1: Confirmation of gene expression results by qRT-PCR for 21 genes (*FOXO1*, *FOXP1*, *GPC4*, *GPSM1*, *IL33*, *INSR*, *KLF2*, *MAN2B2*, *MYO10*, *PAPPA*, *PCSK5*, *PHEX*, *ZFP36*). A strong correlation existed between the microarray and qRT-PCR results (r = 0.9). Table S1: The sequences of 21 primers (forward and reverse) employing B-ACTIN as the endogenous control gene. Table S2: Microarray data for OHSS (PCOS patients with a positive history of OHSS; *n* = 6) and PCOS group (patients with PCOS and have no history of OHSS; *n* = 18) probed using Affymetrix’s GeneChip^®^ Human Genome U133 Plus 2.0 Arrays.
