# Supplementary material for: Global Transcriptional Profiling of Granulosa Cells from Polycystic Ovary Syndrome Patients: Comparative Analyses of Patients with or without History of Ovarian Hyperstimulation Syndrome Reveals Distinct Biomarkers and Pathways
Source: J Clin Med. 2022 Nov 25;11(23):6941. doi: 10.3390/jcm11236941 (PMC9740016; doi:10.3390/jcm11236941)
Supplement: Supplementary file 1 [file jcm-11-06941-s001.zip › Table S1_qRT-PCRprimers.pdf]

Table S1: The sequences of 21 primers (forward and reverse) employing B-ACTIN as the endogenous control gene.

Gene	PRIMERS (5'-3')		Length	Tm (ºC)	Location	Size (bp)	Conc. µM
B-ACTIN	F	CACCACACCTTCTACAATGAGC	22	60.0		157	100
	R	CACAGCCTGGATAGCAACG	19	60.0			
FOXO1-1	F	AAGAGCGTGCCCTACTTCAA	20	60.02	639-661	157	100
	R	CACCCTCTGGATTGAGCATC	20	60.62	806-787		
FOXO3	F	ACAAACGGCTCACTCTGTCC	20	60.31	521-539	168	100
	R	TCTTGCCAGTTCCCTCATTC	20	60.20	670-650		
FOXOP1	F	TCCCACTCTGGGCAACTTAG	20	60.25	1-22	164	100
	R	CTTCAGCTTCCTCTGGATCG	20	60.09	49-28		
IL33-1	F	CAAAGAAGTTTGCCCCATGT	20	59.97	60-80	180	100
	R	AAGGCAAAGCACTCCACAGT	20	59.91	147-125		
PAPPA-1	F	ACAAAGACCCACGCTACTTTTT	22	60.4	494-515	131	100
	R	CATGAACTGCCCATCATAGGTG	22	60.8	624-603		
INSR-2	F	CATCCGGGGATCACGACTG	19	61.9	324-342	45	100
	R	AAGATGACCAGCGCGTAGTTA	21	61.3	368-348		
GPC4-2	F	GTCAGCGAACAGTGCAATCAT	21	61.2	259-279	206	100
	R	ACATTTCCCACCACGTAGTAAC	22	60.0	464-443		
GPSM1-1	F	GGAGCCGGGCCTATCTCTAAA	21	62.9	1725-1745	119	100
	R	CTCTTGCTGCCAGTAAGCATC	21	61.0	1843-1823		
KLF2-2	F	CTACACCAAGAGTTCGCATCTG	22	60.7	846-867	137	100
	R	CCGTGTGCTTTCGGTAGTG	19	60.7	982-964		
MAN2B2-1	F	ACGCCGCCAATGTCTACAC	19	62.7	155-173	92	100
	R	CAGCCGGAAAAACTCCTGCT	20	62.7	246-227		
MYO10-2	F	AAGTGGGGCAGGTAAAACCG	20	62.4	474-493	114	100
	R	GCTCGTTCAACACAGGATGTC	21	61.2	587-567		
NAGA-1	F	TTCCGCTGCAACATTAACTGT	21	60.5	106-126	121	100
	R	TGAGGTATGTGTAGCCCATGTC	22	61.2	226-205		
PCSK5-1	F	CCAACCACTGGGCAGTCAAA	20	62.6	107-126	144	100
	R	CTGACCTTTTAATCGTCCTGCT	22	60.0	250-229		
PHEX-2	F	AAGTCTCCAAGCTAAACAGGAGT	23	60.9	135-157	119	100
	R	AAGCGAACCGGAAGAAATTATCA	23	60.3	253-231		
PLD2-1	F	CAGATGGAGTCCGATGAGGTG	21	61.7	61-81	173	100
	R	CCGCTGGTATATCTTTCGGTG	21	60.3	233-213		
RABGAP1-1	F	ACAGGACTCAAGATTGTAGGGAA	23	60.4	139-161	90	100
	R	CTGGTCGTCCATTGGAGGA	19	60.7	228-210		
SIN3A-1	F	ACCATGCAGTCAGCTACGG	19	61.7	139-157	135	100
	R	CACCGCTGTTGGGTGATGA	19	62.3	273-255		

SQSTM1-2	F	TGCCCAGACTACGACTTGTG	20	61.5	181-200	145	100
	R	AGTGTCCGTGTTTCACCTTCC	21	62.2	325-305		
TCF7L2-2	F	TCCTCGGCAGAGAGGGATTTA	21	61.9	103-123	127	100
	R	CTCGGAAACTTTCGGAGCGA	20	62.2	229-210		
USPX-1	F	TCGGAGGGAATGACAACCAG	20	61.2	29-48	112	100
	R	GGAGTTGCCGGGGAATTTTCA	21	62.9	140-120		
ZFP36	F	GACTGAGCTATGTCGGACCTT	21	61.0	315-335	124	100
	R	GAGTTCCGTCTTGTATTTGGGG	22	60.6	438-417		
