# Supplementary figures and images for: Global Transcriptional Profiling of Granulosa Cells from Polycystic Ovary Syndrome Patients: Comparative Analyses of Patients with or without History of Ovarian Hyperstimulation Syndrome Reveals Distinct Biomarkers and Pathways
Source: J Clin Med. 2022 Nov 25;11(23):6941. doi: 10.3390/jcm11236941 (PMC9740016; doi:10.3390/jcm11236941)

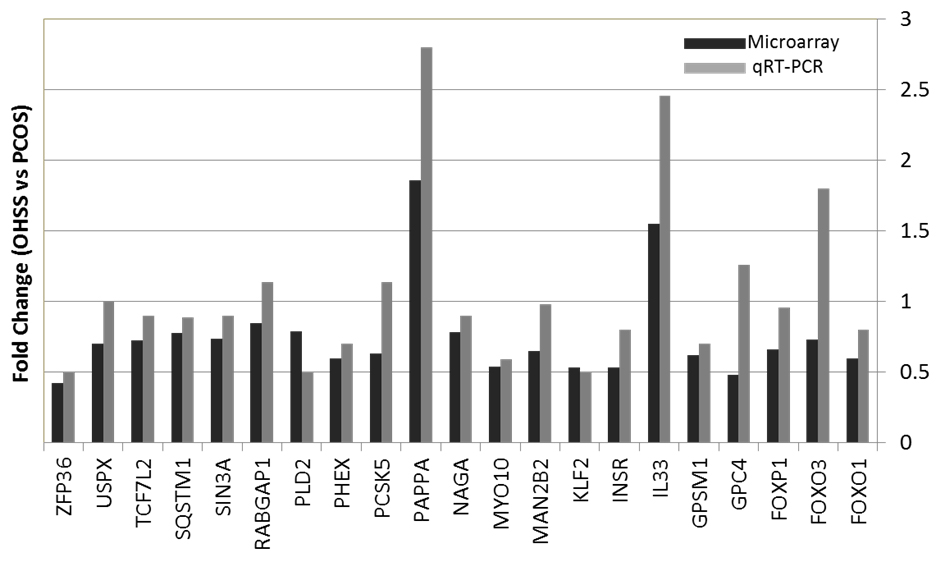

Supplement: Supplementary file 1 [file jcm-11-06941-s001.zip › Figure S1_qRTPCR.jpg]
